# A practical method for the retrieval of tulip-head polyaxial pedicle screw by reusing the rod in revision and implants removal surgery: introduction of technique and evaluation of clinical outcomes

**DOI:** 10.1186/s12893-023-02063-x

**Published:** 2023-06-06

**Authors:** Yao Zhang, Jipeng Song, Yuzheng Lu, Meng Yi, Xiaohang Xu, Lixiang Ding

**Affiliations:** 1 Department of Spinal Surgery, Beijing Shijitan Hospital, Capital Medical University, No. 10, Tieyi Road, Yangfangdian street, Haidian District, Beijing, 10038 People’s Republic of China; 2 Department of Spinal Surgery, Yantai Affiliated Hospital of Binzhou Medical College, No. 717, Jinbu Street, Yantai, Shandong 264000 People’s Republic of China

**Keywords:** Poly-axial pedicle screw, Implants removal, Revision surgery, Hospitalization cost, Bacteria culture

## Abstract

**Background:**

The removal of spinal implants is needed in revision surgery or in some cases whose fracture had healed or fusion had occurred. The slip of polyaxial screw or mismatch of instruments would make this simple procedure intractable. Here we introduce a simple and practical method to address this clinical dilemma.

**Methods:**

This is a retrospective study. The patients underwent new technique for retrieving the implants from July 2019 to July 2022 were labeled as group A, while the patients underwent traditional implants retrieval technique from January 2017 to January 2020 were labeled as group B. Patients in each group were subdivided into revision surgery group (r group) and simple implants removal group (s group) according to the surgery fashion. For the new technique, the retrieved rod was cut off to a proper length which was matched with the size of tulip head, and was replaced into the tulip head. After tightened with nut, a monoaxial screw-rod “construct” was formed. Then the “construct” can be retrieved by a counter torque. The operation duration, intraoperative blood loss, post-operative bacteria culture, hospital stay and costs were analyzed.

**Results:**

A total of 116 polyaxial screws with difficult retrieval (43 screws in group A, 73 screws in group B) in 78 patients were recorded, in which 115 screws were successfully retrieved. Significant differences were found in the mean operation duration, intraoperative blood loss when comparing the r group in group A and B, as well as the s group in group A and B (*P* < 0.05). There were no significant differences in hospital stay and costs between group A and B. Three patients were found positive bacteria culture of drainage tube/tape in group A (3/30), while 7 patients in group B (7/48). The most prevalent bacteria was Propionibacterium acnes.

**Conclusion:**

This technique is practical and safe in retrieving tulip head poly-axial screw. Reduced operation duration and intraoperative bloods loss may potentially alleviate the hospitalization burden of patients. Positive bacterial cultivation results are common after implants removal surgery, but they rarely represent an organized infection. A positive culture with P. acnes or S. epidermidis should be interpreted with caution.

## Background

Surgical stabilization with pedicle screws is a prevalent instrumentation fashion in posterior thoracolumbar and lumbar spine surgeries [1], while fusion is the standard treatment of various spinal conditions, with degenerative causes being the most common indication [2]. The number of spine surgeries performed around the world has increased significantly in recent decades, and the number is only expected to increase [3, 4] Coupled with the increase in primary spine surgery, revision surgery has also followed a progressive increase in numbers [5]. Removal of the implants is an important part during the revision surgery. Moreover, In children, it may be necessary to remove implants early to avoid disturbances to the growing skeleton, to prevent their bony immuring making later removal technically difficult or impossible [6]. In adults, pain, soft tissue irritation, the resumption of strenuous activities or contact sports after fracture healing are typical indications for implant removal in clinical practice [7, 8]. The patients demand of removing the implants was also a kind of “indication”. However, implants removal can sometimes be a difficult task, especially when tulip-head polyaxial pedicle screws (TPS) were included in the instrumentation system. The clinical dilemma we encountered when retrieve the TPS include: 1) mismatch of instruments (patients underwent the primary instrumentation procedure in other medical institution); 2) erosion of the thread of tulip head resulting in screw driver failed to lock the screw; 3) distal head of screw driver broken in the tulip head; 4) incautious manipulation contaminate the screw driver. Those disadvantageous scenario can really annoy the surgeon, as well as the operation duration can be unexpectedly prolonged. Accordingly, we introduce a safe and practical method to retrieve the TPS, in addition, the preliminary clinical outcomes were evaluated in this study.

## Method

### Recruitment of patients

The study was approved by the Ethics Committee of Beijing Shijitan hospital of Capital Medical University, all methods were carried out in accordance with relevant guidelines and regulations. All patients provided written informed consent.

We retrospectively reviewed the medical records and image data of patients from January 2017 to July 2022. The inclusion criteria are as follows: 1) patients with diagnose of thoracolumbar/ lumbar fracture, or degenerative diseases; 2) surgery procedure including the procedure of TPS removal and, in the meantime,  having difficulties during its retrieval; 3) complete medical records and imageology data. Difficulties in implants removal were defined as impossible retrieval of the TPS as the instruments mismatch, damaged screw head thread, or broken of screw driver, and other situations resulting in involuntary discontinuance of surgery. Exclusion criteria include: 1) surgical procedure including screw broken in the pedicle; 2) diagnose including infection or metastasis; 2) incomplete medical records or image data.

#### Patients with new method

From July 2019 to July 2022, thirty patients who underwent the new technique to retrieve TPS and met the inclusion criteria were enrolled. The surgery fashion include revision surgery and single implants removal. This group was labeled as group A, and was subdivided as revision group (Ar group) and single implants removal group (As group).

### Patients with traditional method

From January 2017 to January 2019, forty eight patients met the inclusion criteria who underwent traditional method for TPS retrieval were enrolled. This group was labeled as group B, the subgrouping of which were the same as the group A (Br and Bs subgroup).

The details of patients characteristics were shown in Table 1.

**Table 1:** Demographics and baseline characteristics

	**Total (*n* = 78)**	**Group A (*n* = 30)**		**Group B (*n* = 48)**		***P*-value**
		s group (*n* = 21)	r group (*n* = 9)	s group (*n* = 30)	r group (*n* = 18)	
**Gender**						0.535
Male	40 (53.1%)	11 (52.4%)	4 (44.4%)	16 (53.3%)	7 (38.9.6%)	
Female	38 (46.9%)	10 (47.6%)	5 (55.6%)	14 (46.7%)	11 (62.1%)	
**Age**	39.65 ± 13.08	33.57 ± 8.72	56.33 ± 5.79	30.97 ± 8.55	52.89 ± 5.83	0.151
**Previous Diagnose**						0.312
Fracture	45 (57.8%)	19 (90.5%)	0 (0%)	26 (86.7%)	0 (0%)	
Degenerative disease	33 (42.2%)	2 (9.5%)	9 (100%)	4 (13.3%)	18 (100%)	
**Fixation range**						0.133
Thoracic	4 (5.1%)	2 (9.5%)	0 (0%)	2 (6.6%)	0 (0%)	
Thoracolumbar	38 (48.7%)	11 (52.4%)	3 (33.3%)	17 (56.7%)	5 (27.8%)	
Lumbar/sacral	36 (46.2%)	8 (38.1)	6 (66.7%)	11 (36.7%)	13 (72.2%)	
**Instrumentation construction**						0.165
4-screw	9 (11.5%)	2 (9.5%)	1 (11.1%)	3 (10.0%)	3 (16.7%)	
6-screw	25 (32.1%)	7 (33.3%)	2 (22.2%)	12 (40.0%)	4 (22.2%)	
8-screw	28 (35.9%)	8 (38.1)	4 (44..4%)	11 (36.7%)	5 (27.7%)	
10-screw	9 (11.5)	2 (9.5%)	1 (11.1%)	3 (10%)	3 (16.7)	
12-screw	5 (6.4%)	1 (4.8%)	1 (11.1%)	1 (3.3%)	2 (11.1%)	
14-screw	2 (2.6%)	1 (4.8%)	0 (0%)	0 (0%)	1 (5.6%)	
**Previous surgical procedure**						0.306
Inner fixation only	45 (57.8%)	19 (90.5%)	0 (0%)	26 (86.7%)	0 (0%)	
Include fusion	33 (42.2%)	2 (9.5%)	9 (100%)	4 (13.3%)	18 (100%)	
**Total retrieved screws**	588	160	70	214	144	
**Screws with difficult removal**	116 (19.7%)	28 (17.5%)	15 (21.4%)	41 (19.2%)	32 (22.2%)	0.817

*s group* single implants removal group, *r group* revision surgery group

### Surgical note

#### Group A

**Fig. 1:**
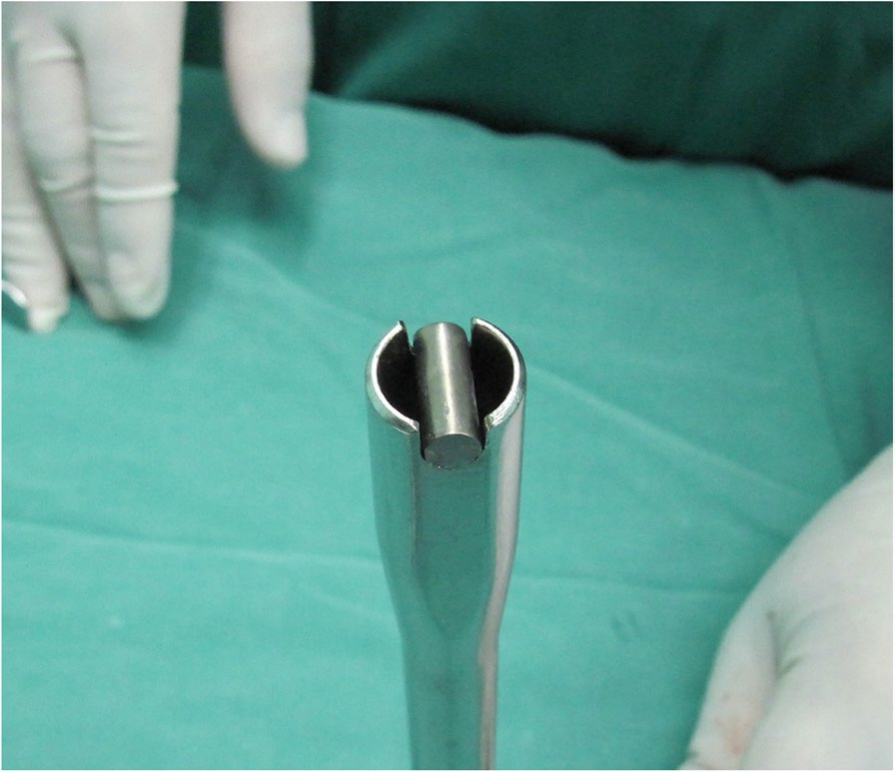
Illustration of new TPS retrieval technique: the rod is reused and is cut off to fit the groove of the counter-torque. A length slightly longer than the groove would be more appropriate

**Fig. 2:**
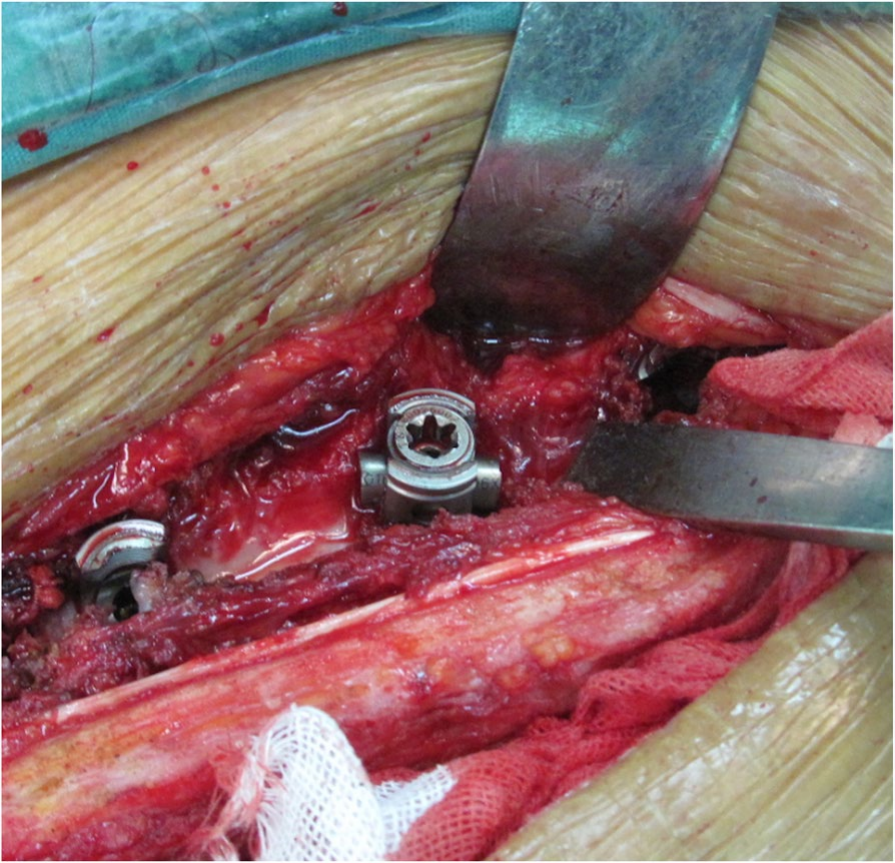
Reset the rod and fasten the nut to form a screw-rod construction. The TPS is transformed to mono-axial screw

**Fig. 3:**
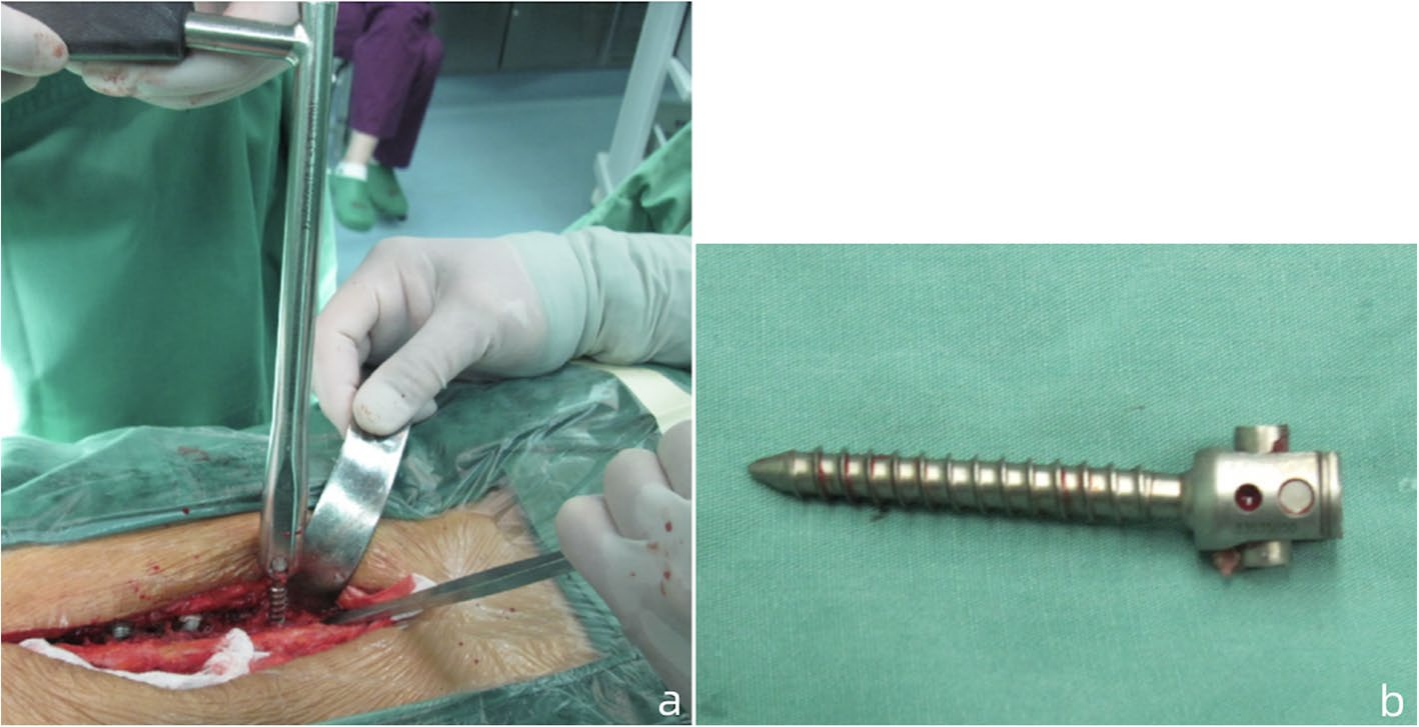
(**a**) The counter torque is used to retrieve the TPS by rotated it in a counter-clockwise direction; (**b**). The retrieved screw-rod construction

In the s group, we followed the previous incision to incise the skin, with the resection of wound scar if necessary. After the subcutaneous tissue was dissected, the deep fascia was exposed. In short segmental fixation (4–6 screws) of thoracic or thoracolumbar vertebrae, the tulip head was superficial. Under this circumstance, we only cut open the deep fascia above the hardware to accomplish the exposure. In long segmental fixation (≥ 8 screws) and fixation of lumbar/sacral vertebrae, the implants were too deep to remove through a small-fascia incision. We usually performed a subperiosteal stripping along the scars to minimize the destruction of paraspinal muscles and hemorrhage. When the difficulties in TPS retrieval occurred, we reused the rod by cutting it off to a fit the groove of the counter torque (slightly longer than the groove) (Fig. 1). The fitted rod and nut were reset to the screw head and were fastened to make the screw and rod become a whole construction (Fig. 2). Subsequently, a poly-axial pedicle screw was transformed to a mono-axial fashion. The screw-rod construction can be retrieved by using the counter torque (Fig. 3a, b).

In the r group, the fashion of the implants exposure was the same as that in long-segmental fixation of s group. The detailed revision procedure was not described here.

Before suture the incision, we dwelled a drainage tube or drainage tape in r group and s group, respectively.

#### Group B

The principle of exposing the implants was the same as the above mentioned in group A.

**Fig. 4:**
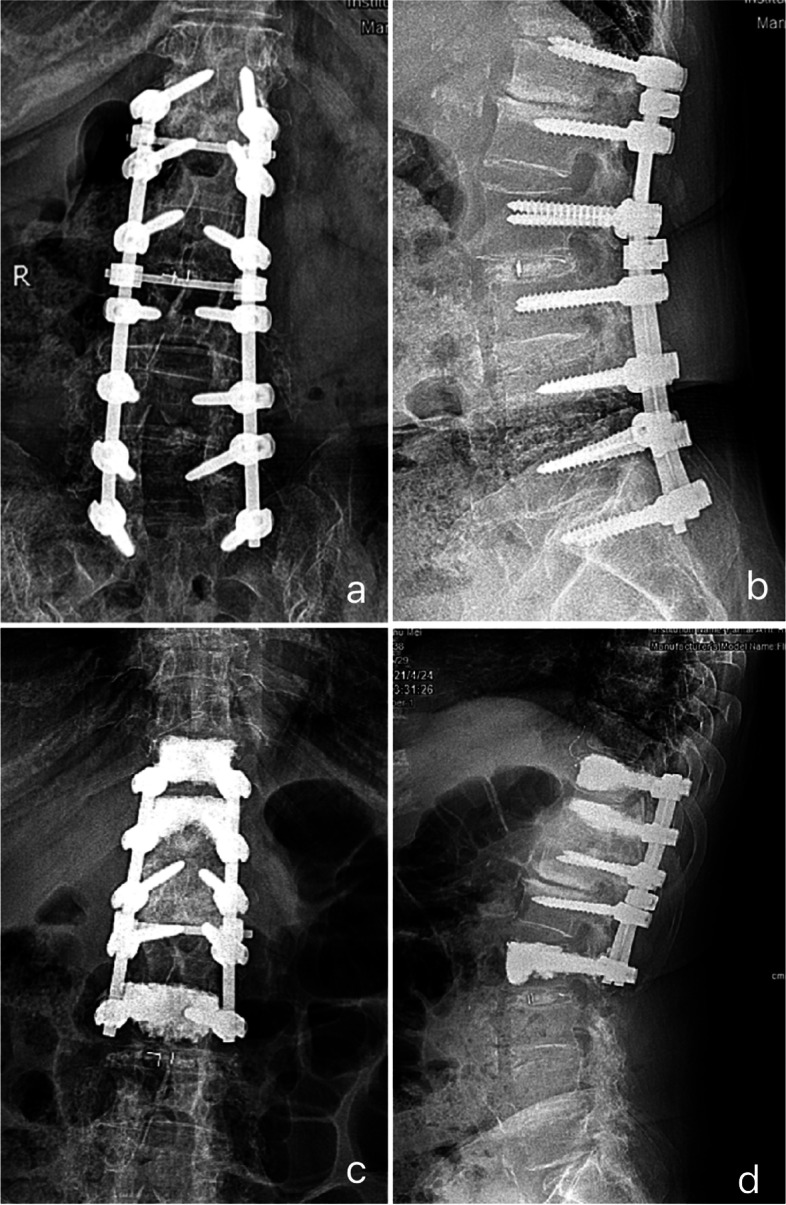
Case presentation in Br group.** a**). Anteroposterior view of a 45-year-old female patient underwent instrumentation from S1 to T12, posterolateral fusion from S1-L3, and interbody fusion of L2/3 five years ago; (**b**). Lateral view demonstrate the plow-out of screws in T12; (**c**), (**d**). We encountered difficulties when retrieving the bilateral L2 and L1 TPS. On account of large-diameter screw failed to provide enough pull-out strength in L2, bone cement was used to augment the screws. Bone cement augmentation was also applied to T10 and T11 to ensure the overall constructive stability

When the difficulties in TPS retrieval occurred, a small round-head burr was used to slightly enlarged the screw canal. In most cases, enlargement of superior 1/3 to 1/2 part of screw canal could make the screw retrieval feasible. Always kept in mind that the iatrogenic fracture of the pedicle should be avoided. In the s subgroup, an 18-year-old male patient who had previous L3-T10 TPS instrumentation was failed to remove the right-side L1 screw on account of the osteosclerosis of screw canal and congenital narrowed pedicle. The screw were left in situ. In the r group, a 45-year-old female patient underwent revision surgery for adjacent segmental disease. We encountered difficulties in retrieving the bilateral L1 and L2 screws as the erosion of the thread of screw head. We enlarged the screw canal to successfully removed the screws. However, screw loosening occurred in L2 even we increased the screw diameter. Consequently, the bone cement was applied to augment the screw (Fig. 4).

### Postoperative management

Prophylactic antibiotics were routinely applied in r group until one day after surgery. The drainage tube were removed within 48 h—72 h in r group. In s group, drape changing was performed on first postoperative day and drainage removal was performed on second postoperative day. Bacteria culture of the distal end of the tube and tape were routinely performed to assist to evaluate the deep surgical site infection. The application of therapeutic antibiotic depend on the results of bacteria culture and the subsequent antibiotic susceptibility. The inflammatory markers including ESR (erythrocyte sedimentation rate), C-RP (C-reaction protein) and leukocyte counts were followed during hospitalization, which will decide the course of antibiotic application. The indication of discharge include good general condition with a normal temperature, significant downtrend of inflammatory markers and well-healed wound. Suture removal can be performed outside the hospital.

### Statistical analysis

All data were statistically analyzed using SPSS software (version 25, SPSS Inc, Chicago, Illinois). Continuous variables were described by means and standard deviations, while categorical variables were described by frequencies and percentages. Differences between groups were analyzed using chi-square tests. Student's t-test for independent samples was used to compare normally distributed data between two groups. Categorical variables were assessed using chi-square tests and Fisher's exact tests. A *p* < 0.05 was considered statistically significant.

## Results

A total of 78 patients with a mean age of 39.65 ± 13.08 (range: 17–68) years were finally analyzed. The demographics, previous diagnose, fixation range, previous surgical procedure, the numbers of retrieved screws and so on are shown in Table 1. No significant differences were found in demographics, and others baseline characteristics when comparing group A and group B (*p* < 0.05, Ar vs Br, As vs Bs, respectively).

The mean operation duration, intraoperative blood loss, hospital stay, hospitalization costs of the two groups were shown in Table 2. Significant differences were found in operation duration and intraoperative blood loss when comparing the As group with the Bs group, as well as comparing the Ar group and Br group (*p* < 0.05). No significant difference was found in hospital stay and costs when comparing between the corresponding subgroups.

**Table 2:** Comparison of operative and postoperative data between subgroups

	**Ar group**	**As group**	**Br group**	**Bs group**	**P**^**1**^	**P**^**2**^
Operation duration (min)	149.67 ± 35.08	57.71 ± 10.14	224.72 ± 41.39	109.07 ± 12.00	***p* < 0.001**	***p* < 0.001**
Intraoperative blood loss (ml)	570 ± 170.07	125.67 ± 47.12	762.22 ± 131.80	257.97 ± 98.89	***p* < 0.001**	***p* < 0.001**
Hospital stay (day)	13.11 ± 3.06	7.67 ± 2.46	12.17 ± 2.87	7.50 ± 2.13	*p* > 0.05	*p* > 0.05
Hospitalization costs (CNY)	72,532.56 ± 11,161.40	8862.76 ± 1904.68	69,093.61 ± 15,604.07	8845.63 ± 1830.41	*p* > 0.05	*p* > 0.05

*As* s subgroup of group A, *Ar* r subgroup of group A, *Bs* s subgroup of group B, *Br* r subgroup of group B, *P*^*1*^ comparison between As and Bs, *P*^*2*^ comparison between Ar and Br

With regard to the postoperative bacteria culture, three patient (10.0%) manifested positive results in group A, while seven patients (14.6%) in group B. The total positive culture rate was 12.8%, with 22.2% in the revision group and 7.8% in the implants removal group. The time interval from drainage removal to culture results report was 4–7 days. The results of bacteria culture and antibiotic application were shown in Table 3.

**Table 3:** Patients with a positive bacteria culture result; application of antibiotic and course

	**Bacteria**	**Ab**	**Medication method**	**Duration of Ab application**
As 1	S. epidermidis	vancomycin	iv drip	7 days
Ar 1	P. acnes	levofloxacin	PO	5 days
Ar 2	Staphylococcus. aureus	linezolid	iv drip	7 days
Bs 1	S. epidermidis	none	none	none
Bs 2	P. acnes	sulperazone	iv drip	5 days
Bs 3	P. acnes	moxifloxacin	PO	10 days
Br 1	Staphylococcus. aureus	vancomycin	iv drip	6 days
Br 2	P. acnes	none	none	none
Br 3	S. epidermidis	PSTS	iv drip	8 days
Br 4	P. acnes	levofloxacin	PO	10 days

*S. Epidermidis* Staphylococcus. Epidermidis, *P. acnes* Propionibacterium acnes, *Ab* antibiotic, *PSTS* piperacillin sodium and tazobactam sodium, *iv drip* intravenous drip, *PO* per OS

## Discussion

Rates of spine surgery have increased dramatically over the past decade [3, 4, 9]. The increased use of internal fixation to treat spinal diseases has enhanced interest in implant removal surgery [10]. Previous study have shown that implant removal surgery contributed to almost 30% of all planned orthopaedic operations, and 15% of all operations of the department [10]. In children and adolescent patient, it may be necessary to remove implants early to avoid disturbances to the growing skeleton, or to prevent their bony immuring making later removal technically difficult or impossible [11]. In addition, implants removal after fracture has healed or fusion has achieved are beneficial to regain range of motion for manual workers. However, Hanson, et al. [6] studied the surgeons' practice and perceived effectiveness of implant removal. The results showed a part of surgeon do not believe in clinically significant adverse effects of retained metal implants. Nevertheless, adverse effects of indwelling metal like stress shielding, allergic or carcinogenic potential are perceived by a considerable portion of surgeons, and even patients [12]. The research regarding the indications and adverse effects of spinal implants removal is scanty. Each hospital should establish it’s strict criteria for implant removal. Xu et al. [13] reported the necessity of implants removal after fixation of thoracolumbar and lumbar burst fractures without fusion in elderly patients. They found that local range of motion increased after implants removal. Jeon et al. [14] demonstrated that pedicle screw removal after successful posterior fusion of thoracolumbar burst fractures is beneficial because it alleviates pain and disability, moreover, restoration of the segmental motion angle after implant removal may contribute to the clinical improvement. Stavros et al. [15] studied 57 patients who have undergone removal of pedicle screws because of pain and discomfort with fracture being the initial diagnosis in 40% of the patients, and degenerative spine disease being the initial diagnose in 58%. They found removal of pedicle screws may be effective in alleviating back pain. In our institution, except for the necessary implants removal in revision surgery group, the patients in the simple implant removal group are relative young (33.57 ± 8.72 years old in As, 33.57 ± 8.72 years old in Bs; the eldest: 46 years old). In our clinical practice, younger patients are tend to remove their implants of one's own accord. However, the advantages and disadvantages after implants removal are beyond the scope in this study.

In 1959, Boucher et al. [16] introduced the pedicle screw fixation technique in spinal fusion. Multiaxial pedicle screw allow deviation of the screw away from the perpendicular to the longitudinal rod, which facilitates application of a screw–rod system into the curved spine [17, 18]. The ball-in-cup mechanism allow the screw to be locked in a tilted position. However, given the locking mechanism, the removal of TPS is more difficult than mono-axial screw. In clinical practice, it is frequent that the primary surgery and revision/simple implants removal surgery are performed in different medical institution. Consequently, the surgeon may be unfamiliar with the type of previous implants or does not have a suitable instrument to remove the implants as the matched instruments are no longer produced. Moreover, conditions such as the erosion or iatrogenic destruction of the screw thread, screw driver distal head broken or unexpected contamination of device, are able to make the procedure reach an impasse. Some researcher have provided their solution. Kamil et al. [19] bend the retrieved rod into “U” shape. One arm of the “U” shape rod (as short as possible in order to prevent any destruction to the surrounding structures during screw retrieval) was reset into the tulip of screw and was then tightened with nut, then the screw can be removed with a plier by holding the other end of the rod and rotating the rod-screw complex in a counterclockwise direction. Our technique is easier in comparison of Kamil’s. First, cutting out a short rod is easier than reshaping an U shape rod. Harvesting an rod with suitable length that fit the tulip head is not simple, an overlong rod arm may result in soft tissue obstruction during the retrieval process. Second, the two ends of the U shape rod were in a straight line, screw driver was unavailable because it can be obstructed. Consequently, they used an Allen wrench to tighten the nuts, however, the arm of force might be too short to completely lock the system. Moreover, the locking process can be difficult in case the screws were deeply located. Finally, if the rod removal procedure was obstructed, this technique is infeasible. In contrast, in our technique, if we got stuck in rod removal procedure at the beginning, a saw or lock wrench was used to cut the rod near the screw head, leaving the nut in situ to form the screw-short rod construction. However, the patients with broken pedicle screw were not included in this study, as quite a few studies have reported resolution for this clinical scenario [20–23].

It is generally recognized that the implants removal should be a quick and safe procedure. However, the unexpected prolonged operation duration during implants retreival not only annoy the surgeon, but increase the anxiety of patients [24]. In this study, we found that the operation duration and intraoperative blood loss were significantly reduced in group A in comparison of group B, it mainly attributes to the easy retrieval of implants. However, according to the present results, there are no significant difference in hospital stay and total costs between group A and B. It might be that no extra consumption was generated during the implants removal procedure. Moreover, the factors that affected the hospital stay are relevant to the main revision step and perioperative complications. The highest hospitalization costs occurred in the patient with unpredictable bone cement remedy in subgroup Br (107, 863 CNY versus mean costs 69093.61 ± 15604.07 CNY). A significant difference of hospitalization costs might occur if the sample size was larger.

We also studied the microbiology at deep surgical site through postoperative drainage tube/tape culture. The most prevalent bacteria was P. acnes, followed by S. epidermidis. P. acnes is a slow-growing, aerotolerant, anaerobic gram-positive bacteria that has been linked to discitis, spondylodiscitis, osteomyelitis and paravertebral infection following surgical procedures [25, 26]. Plenty of studies have discussed the relationship between P. acnes and retrieved implants. Leitner et al. [27] presented cultivation results from 110 cases of spinal metal implants and identifed positive bacteria culture in 29% of cases, most frequently Staphylococcus (53.1%) and Propionibacterium (40.6%). They found patients with screw loosening had significantly higher rates of positive cultivation. Callanan et al. [28] presented an uncontrolled case series on 43 patients with no signs of infection undergoing revision surgery after previous instrumentation. They cultivated bacteria in 37% of cases with P. acnes being the most prevalent bacteria. According to Søren et al. [29], they included 32 pseudarthrosis and 32 controlled patients who underwent revision surgery. Interestingly, they concluded that pseudarthrosis after instrumented spinal surgery was not signifcantly associated with the presence of bacteria at the pseudarthrosis site. Positive cultivation results are common after spinal instrumentation, but they rarely represent an organized infection. We routinely performed the bacteria culture of postoperative drainage tube or tape, rather than the implants. The positive culture rate is 12.8%, with 22.2% in the revision group and 7.8% in the implants removal group, which is much lower than the results from others studies. Moreover, the diagnose of postoperative infection was not valid in patient with a positive bacterial culture result. Gelalis et al. [30] found a contamination rate of 20% of patients after randomly cultivating wound swaps. Since Propionibacterium acnes is a commensal organism of low virulence that populates the dermal sebaceous glands, positive cultures are difficult to interpret in patients with no obvious signs of infection [31]. However, a positive culture with P. acnes should be interpreted with caution. In this study, therapeutic antibiotic regimen was likely to be applied to handle this scenario, which depend on the clinical manifestation, antibiotic susceptibility results and variation tendency of inflammatory markers (mainly C-RP, ESR, leukocyte counts). Contamination of specimen (e.g. during the drainage removal) should be considered, especially in patient with good general condition, normal temperature, significant downtrend of inflammatory markers and well-healed wound.

There are several limitations in this study. Firstly, our sample size is small, involving only 78 patients. Although the main purpose of this study is to introduce a new technique, and a satisfactory outcome has obtained in the present study, larger sample sizes will be needed to provide stronger evidence for our conclusions. Secondly, we use the postoperative drainage as the culture rather than using the implants, the positive culture results might not be able to fully interpret the deep surgical site infection. Nevertheless, this culture method is easy to perform, in addition, the results could play a role as the hint of infection. Finally, the patients should be followed after discharge as delayed-onset infection can occur at a very late time.

## Conclusion

The described technique was practical and convenient in handling difficult TPS retrieval. Reduced operation duration and intraoperative bloods loss may potentially alleviate the hospitalization burden of patients. Positive cultivation results are common after spinal implants removal surgery, but they rarely represent an organized infection. A positive culture with P. acnes or S. epidermidis should be interpreted with caution.

## Data Availability

All data used by or generated in this study is available from the corresponding author upon reasonable request.

## Abbreviations

TPS: Tulip-head poly-axial pedicle screw
C-RP: C-reaction protein
ESR: Erythrocyte Sedimentation Rate
P. acnes: Propionibacterium acnes
S. epidermis: Staphylococcus. Epidermidis
Ab: Antibiotic
